# Extremely Rare Case of Cavernous Haemangioma of Submandibular Gland 

**DOI:** 10.22038/IJORL.2022.64705.3221

**Published:** 2022-11

**Authors:** Rishita Kalra, Sama Rizvi, Vivek Kumar Pathak, Pradeepti Nayak

**Affiliations:** 1 *Department of Otorhinolaryngology,* *School of Medical Sciences and Research, Sharda University, Greater Noida, Uttar Pradesh, India.*

## Abstract

**Introduction:**

Haemangioma or hemangioendothelioma is amongst the commonest developmental, vascular lesions of infancy and childhood. Hemangioendothelioma of the salivary glands, however, is extremely rare. Due to their rarity, they may be misdiagnosed as lymphangiomas or other cystic lesions found more commonly in the region. This may lead to surgical complications including torrential hemorrhage that may have dire consequences for the patient.

**Case Report:**

Herein we present the case of a nine-year-old male with a cavernous haemangioma involving the left submandibular gland which was diagnosed on-table due to inconclusive pre-operative radiological and pathological diagnosis. Fortunately, due to meticulous dissection and care the lesion was excised in toto without any significant blood loss.

**Conclusions:**

Haemangioma of the submandibular gland is so uncommon that often it isn't even considered a differential diagnosis for cystic swellings and lesions in this region. Mistaken diagnoses preoperatively may prove disastrous for the patient. Excision of haemangiomas requires planning for hemostasis and blood loss if it occurs. Even minor iatrogenic manipulation of vascular lesions may completely obscure the field due to bleeding, making dissection and recognition of anatomical landmarks very difficult. This is especially dangerous in the submandibular region due to the proximity of various vital vascular and neural elements. A differential of haemangioma should therefore always be considered by surgeons and radiologists alike for lesions with suspicious or indeterminate features, in this region.

## Introduction

Haemangiomas are amongst the commonest benign lesions. However, salivary gland haemangiomas are very rare (1,2). Unless there is a high degree of suspicion, haemangiomas of salivary glands are often misdiagnosed due to their rarity and unusual histopathological features. Salivary gland hemangiomas represent less than 5% of all salivary gland tumors of which 90% are found in the parotid gland. Haemangiomas generally present with skin lesions characterized by vascular blush, engorged vessels, or pulsations on palpation of the submandibular region. Imaging may reveal phleboliths which are very suggestive of a vascular malformation. In the absence of these signs, the diagnosis can be difficult, since a hemangioma in the submandibular gland is very uncommon (3).

## Case Report

A nine-year-old male presented to the ENT OPD with the chief complaint of a left-sided neck swelling of three months duration. It was associated with facial disfigurement and restricted neck movements. The informant (father) revealed that the swelling was insidious in onset and gradually increased in size. Initially, two pea-sized swellings were observed which then coalesced and grew to attain the current size. It was painless and not associated with fever, weight loss, or malaise. There was no history of difficulty swallowing or breathing. There were no relevant co-morbidities. 

It was diagnosed as a case of lymphangioma after contrast-enhanced CT of the neck at a hospital elsewhere two months previously. Two doses of intra-lesional bleomycin (3IU/mL) were administered six weeks apart. After the first dose, the swelling reduced in size. After the second dose, however, the swelling increased in size and the consistency changed from soft to firm. FNAC was repeated at the same hospital which was then suggestive of hematoma. 

**Fig 1:**
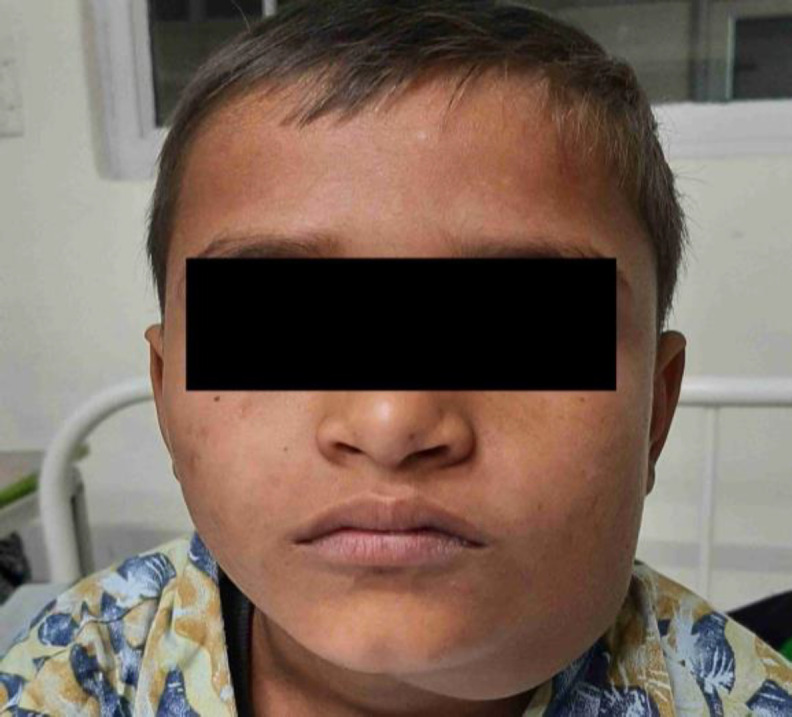
Front view showing the left submandibular swelling

On presentation at our hospital, local examination revealed a solitary, 6*5 cms ovoid, firm, mobile, non-compressible, non-reducible, non-tender mass extending from the left angle of the mandible laterally to midline medially. It extended up to the hyoid bone inferiorly, overlying the ramus of the mandible, and occupied the entire submandibular region (Figure 1). 

The margins were regular. The transillumination test was negative. The overlying skin and local temperature were normal. On oral cavity examination, the left tonsillar region was pushed medially. The floor of the mouth was found to be elevated. The lesion was bimanually palpable. There was no evidence of cervical lymphadenopathy.

Routine blood investigations were within normal limits. The Mantoux test was negative.

**Fig 2:**
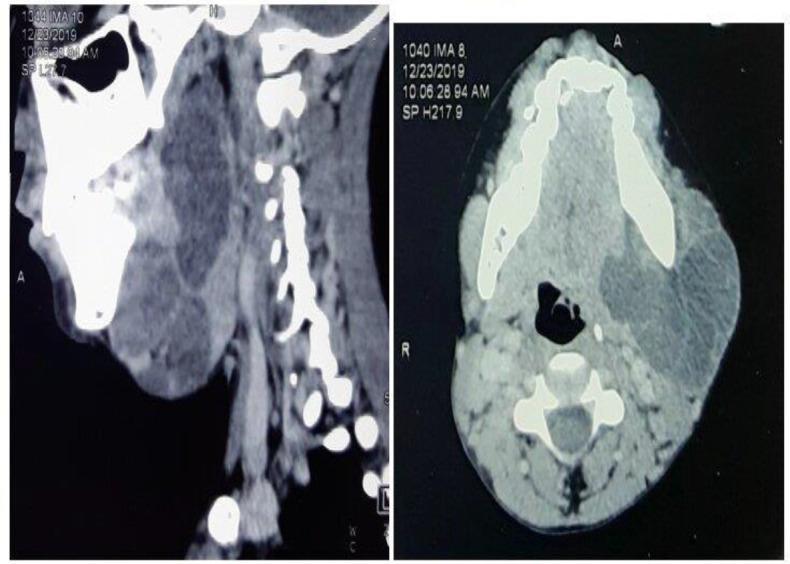
Sagittal and Axial sections showing the multi-septate cystic lesion with extension to the para-pharyngeal region

Contrast-enhanced CT of the neck revealed a large, lobulated, multi-septate, cystic lesion in the left submandibular gland extending into the left para-pharyngeal space. Superiorly it extended up to the nasopharynx, the left parotid region, and masseter muscle and inferiorly to the sublingual space (Figure 2).

There was evident compression of the oropharyngeal airway with rightward displacement. There was no calcification or underlying bone changes. 

**Fig 3:**
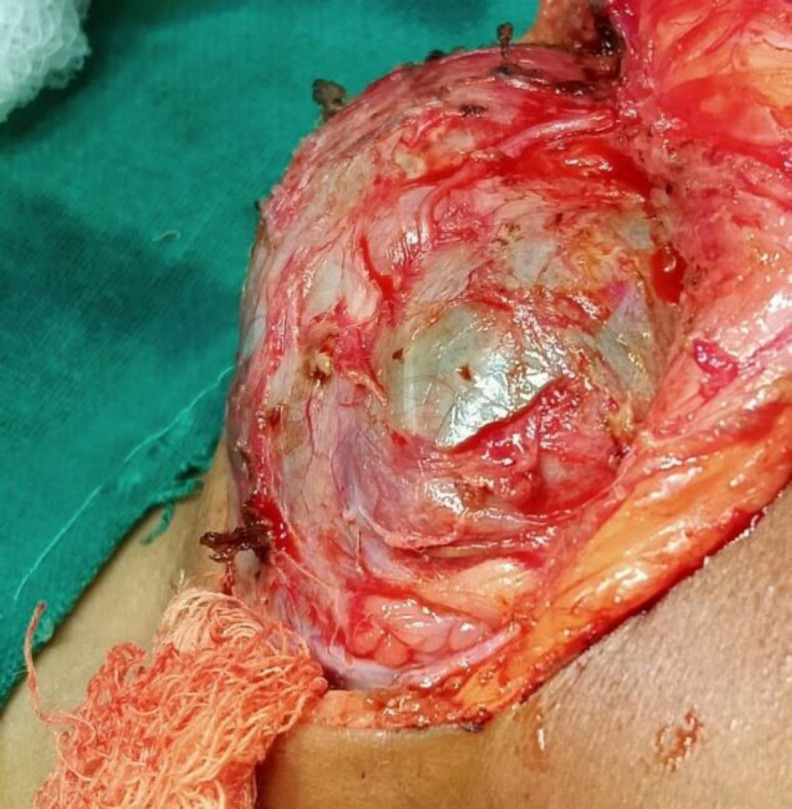
Intraoperative picture of the vascular mass within its capsule

The patient was taken up for surgery after appropriate pre-anesthetic clearance and written and informed consent. Under general anesthesia with orotracheal intubation, the subcutaneous tissue, fat, and fascia over the lesion were carefully dissected. (Figure 3).

The marginal mandibular nerve was found to be splayed and partially embedded in the capsule of the lesion. It was carefully separated and retracted superiorly. During the dissection one locule of the lesion burst and dark red, fresh blood spilled out, leading us to suspect that this may in fact be a vascular lesion. The capsule extended from the skull base to the hyoid bone and was carefully separated by blunt and sharp dissection. The facial artery, facial vein, and submental artery were identified and ligated. The hypoglossal nerve and lingual nerve were identified and preserved. The lesion was excised in toto and sent for histopathological examination. The cumulative blood loss during the procedure was around 200 ml. 

Histopathological examination showed a single globular tissue, measuring 6*6*3 cm with multiple cysts. On sectioning, it yielded hemorrhagic fluid and coagulated blood.

**Fig 4:**
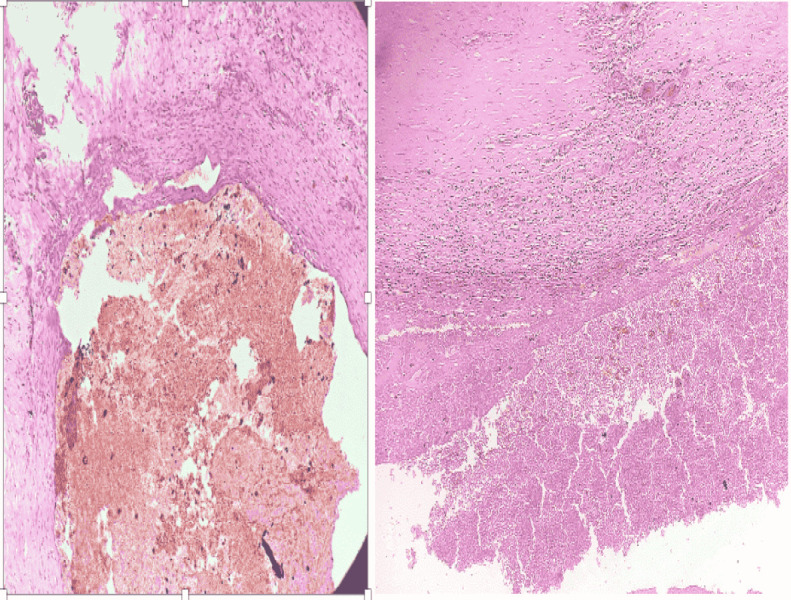
Microscopic pictures showing thin-walled blood vessels, dense lymphoplasmacytic infiltrate, and normal salivary gland tissue with areas of hemorrhage

Microscopic examination showed cystically dilated, endothelium-lined blood vessels filled with intact and lysed red blood cells and fibrin deposition. Surrounding tissue had few thin-walled blood vessels, dense lymphoplasmacytic infiltrate, skeletal muscle bundles, and normal salivary gland tissue with areas of hemorrhage. These features were diagnostic of cavernous hemangioma of the submandibular gland (Figure 4).

Post-operatively, the patient was asymptomatic with minimal serosanguinous discharge in the drain over 24 hours. Intravenous antibiotics and analgesics were administered. There were no features of facial nerve palsy or any other complications. The patient was discharged on postoperative day seven. On follow-up visits at one week and six months, the patient was asymptomatic with complete relief from the pre-operative symptoms.

## Discussion

Hemangiomas are vascular abnormalities that are characterized by increased proliferation and renewal of endothelial cells (4). Haemangiomas are considered congenital benign neoplasms. They present at birth or during childhood and grow progressively till the patient reaches adolescence. They have a female predilection and present more commonly on the left side (5).

It encompasses three distinct clinical entities - capillary hemangioma, cavernous hemangioma, and mixed hemangioma. Compared to capillary hemangioma, cavernous hemangioma is rare and tends to occur in older children and adults (5). Also unlike capillary haemangiomas, cavernous haemangiomas do not generally regress with time. 

The parotid gland is the most common salivary gland involved. Childers et al found only ten cases of salivary gland haemangiomas in a 30-year retrospective study of all cases of haemangiomas retrieved from the Registry of Oral and Maxillofacial Pathology of the Armed Forces Institute of Pathology at Washington DC, all of which were located in the parotid gland (6). In another study, 779 reported cases of salivary gland tumors were evaluated during 25 years in a single hospital. Of these only three cases of hemangiomas were reported in the parotid gland and none in the submandibular gland (2). 

 Hemangioma of the submandibular gland is extremely rare. Our PubMed and Medline search of English language reports revealed only 17 cases of submandibular haemangioma. Of these, eleven cases occurred in females and six cases were seen in males. (Table1, Table 2) The patients ranged from 7–65 years and the average was 36 years (7-17). 

**Table 1:** Reported Cases of Submandibular Gland Cavernous Haemangioma- Clinical Features

	**Study**	**Sex/ Age**	**Fluctuation**	**Skin Changes**	**Pain**	**Postprandial increase in Size**
1	Lee HJ et al^16^	F/34	No	No	No	No
2	Cho et al^10^	F/41	Yes	No	No	No
3		F/16	Yes	No	No	No
4		F/49	Yes	No	No	No
5		M/44	Yes	No	Yes	No
6		M/57	Yes	No	No	No
7	El Hakim IE et al^13^	F/35	No	No	Yes	No
8	McMenamin et al^14^	F/37	Yes	No	Yes	Yes
9	Chuang et al^12^	M/65	Yes	No	Yes	No
10	Ozturk et al^9^	M/27	Yes	No	No	No
11	Kumar et al^11^	M/20		No	Yes	No
12		F/35		No	Yes	No
13		F/47		No	Yes	No
14		F/37		No	Yes	No
15	Azadarmaki et al^7^	F/52			No	
16	Bowerman et al^15^	F/16		No	Yes	No
17	Aynali et al^8^	M/7		No	No	
18	Sasaki et al^17^	F/31		No	No	

**Table 2:** Reported Cases of Submandibular Gland Cavernous Haemangioma – Investigations and Management

	**Study**	**Sex/ Age**	**USG**	**CT**	**MRI**	**FNAC**	**Management**
1	Lee HJ et al^16^	F/34	Hypoechoic	Minimal Enhancement		Inconclusive	Excision of the mass with Submandibular gland excision
2	Cho et al^10^	F/41				Inconclusive	Excision of the mass with Submandibular gland excision
3		F/16				Inconclusive	Excision of the mass with Submandibular gland excision
4		F/49				Inconclusive	Excision of the mass with Submandibular gland excision
5		M/44				Inconclusive	Excision of the mass with Submandibular gland excision
6		M/57				Inconclusive	Excision of the mass with Submandibular gland excision
7	El Hakim IE et al^13^	F/35	Hypoechoic	Enhancement and calcification		Inconclusive	Excision of the mass with Submandibular gland excision
8	McMenamin et al^14^	F/37		Minimal Enhancement and calcification		Inconclusive	Excision of the mass with Submandibular gland excision
9	Chuang et al^12^	M/65		Calcification		Inconclusive	Excision of the mass with Submandibular gland excision
10	Ozturk et al^9^	M/27	Anechoic	Cystic	Cystic, hypointense on T1	Inconclusive	Excision of the mass with Submandibular gland excision
11	Kumar et al^11^	M/20		Enhancement		Inconclusive	Excision of the mass with Submandibular gland excision
12		F/35		Enhancement		Inconclusive	Excision of the mass with Submandibular gland excision
13		F/47		Enhancement		Inconclusive	Excision of the mass with Submandibular gland excision
14		F/37		Enhancement		Inconclusive	Excision of the mass with Submandibular gland excision
15	Azadarmaki et al^7^	F/52		Non-enhancing mass with an enhancing vessel (periphery)		Inconclusive	Submandibular gland excision with dissection of Level IB lymph nodes
16	Bowerman et al^15^	F/16				Inconclusive	Excision of the mass with Submandibular gland excision
17	Aynali et al^8^	M/7		Heterogeneous hypodense areas with calcified foci		Inconclusive	Excision of the mass with Submandibular gland excision
18	Sasaki et al^17^	F/31		Phlebolith	Isointense on T1 and Hyperintense on T2		

Reports where the gland was completely absent, as in the cases reported by Hopkins et al and Iguchi et al, are not included amongst these. Also, submandibular gland haemangioma extending to para-pharyngeal space is even more unusual with only one previously reported case with similar manifestations (7). Ours is only the second case to report this finding. Cavernous haemangioma is histo-pathologically characterized by variable, dilated, thin-walled, vascular spaces lined by flattened endothelial cells without atypia. They are usually enclosed by a collagen layer. The vessels may have a lobular arrangement or maybe diffuse with no distinctive pattern. The walls may occasionally be thickened due result of fibrosis of the adventitia. Inflammatory cells may be seen in the stroma as observed in this case (11). Other vascular masses in the submandibular region include intramuscular hemangioma, lymphangioma, and angiosarcoma. Though much more common, lymphangiomas rarely arise from salivary tissue. Intramuscular haemangioma and angiosarcomas of the submandibular gland are even more uncommon (18-20). 

Venous haemangioma and arterio-venous malformations have also been reported in the submandibular gland. However, very few cases have been reported so far of such venous malformations (21-23).

90% of all haemangiomas are seen in children and young adults (24). Nagao et al, in their series of 20 patients with parotid haemangioma found that though the ages of the patients ranged from four months to 50 years, the mean age of the patients was 26 years, making it most common in the young adult age group (25).

Most of the infantile haemangiomas in salivary glands are capillary haemangiomas. Only the cavernous variant is generally found in adults. One case of submandibular cavernous haemangioma has been reported previously in a young child (8). In this case, the patient was a seven-year-old boy with para-pharyngeal extension making it the first ever reported case with such findings. Concerning gender distribution, submandibular haemangiomas are more commonly encountered in females with a ratio of 2:1. It is believed that the proliferating endothelium in these vascular tumors is stimulated by circulating hormones. This theory is given traction by the fact that studies have reported an increase or fluctuation in the size of these lesions during menarche and pregnancy (26). Unlike haemangiomas in other areas, those in salivary glands like parotid and submandibular glands, being deep-seated, may not present with the characteristic vascular blush or engorged vessels. The clinical presentation of a haemangioma is usually that of a slow-growing, soft, painless mass. There may be pain or a sudden increase in size in case of hemorrhage into the surrounding tissues or recurrent inflammation (15). 

Recurrent inflammation may also lead to mass being firm on palpation, due to subsequent fibrosis (16). Episodic increase in the size of the swelling associated with pain may erroneously lead to the suspicion of sialadenitis or sialolithiasis (11,12,14,27). Haemangiomas are generally characterized by the presence of phleboliths. Phleboliths are thrombi that form due to turbulent flow in the vascular channels and harden over time due to the deposition of calcium within it. It is generally seen in long-standing venous or capillary vascular lesions where the blood flow is sluggish. Phleboliths may, contrarily, also complicate the diagnostic evaluation. Cases have been reported of cavernous haemangiomas with phleboliths presenting as sialolithiasis in the absence or aplasia of the gland itself (27,28). In such cases, sialography can help differentiate between phleboliths and sialoliths. With respect to the vasculature, the facial and lingual arteries have been recognized as the most common feeding vessels, when identified (11). Radiography, Ultrasonography, 99mTc RBC Scintigraphy, Xeroradiography, Computed tomography (CT), CT Angiography, and MRI and MRI STIR are some of the radiological modalities that may assist in the evaluation of vascular lesions like haemangiomas (29). Ultrasonographically, cavernous haemangiomas in salivary glands generally have well-defined borders and heterogeneous or homogeneous echogenicity with or without phleboliths or calcification (29). Additionally, their greyscale sonographic appearance has been reported to be nonspecific (30). The detection of blood vessels in hemangiomas may be difficult. However, haemangiomas may show the presence of phleboliths, which may confirm the diagnosis. Studies have reported that plain film X-rays demonstrate calcified phleboliths in 2–3% of cases (26). Ultrasound, like radiographs, can also reliably detect hemangiomas with phleboliths. Duplex or doppler sonography may better differentiate vascular lesions from other cystic lesions. However, it detects only the blood flow to the lesion and not the extent of the vascularity. Nevertheless, doppler ultrasonography should be considered an essential part of the evaluation to detect the presence and extent of vascularity of such lesions (31). 99mTc RBC scintigraphy is historically considered pathognomonic for vascular lesions. 

Studies have shown that the specificity and sensitivity of 99mTc scintigraphy are as high as 100% and 89% respectively for cavernous haemangioma (29). These days CECT with angiography has replaced scintigraphy as the gold standard for the diagnosis of haemangiomas. Contrast-enhanced CT with angiography is considered the imaging modality of choice for haemangiomas. CT scans generally show hemangioma as an enhanced, well-circumscribed, lobulated mass with homogeneous density. MRI is also useful in evaluating hemangiomas and their extensions (32). On an MRI image, hemangiomas typically appear as well-defined, lobulated, and homogeneously enhancing lesions with homogeneous T1isointensity with muscle and T2 hyperintensity with variable vascularity. Enlarged vessels may be seen as signal voids (33).

 FNAC is often inconclusive and reveals only blood components presenting as a hematoma. In fact, in all the reported cases of submandibular haemangioma, the FNAC was inconclusive. An indeterminate FNAC with radiological features of a vascular pathology should raise the suspicion of a haemangioma. If a vascular lesion is suspected early on, FNAC should be avoided to prevent iatrogenic trauma and hematoma formation. 

Because cavernous hemangiomas tend to be larger, less well-circumscribed than capillary hemangiomas, and show no tendency to regress, surgery remains the management modality of choice. In all the previously reported cases the lesions were surgically excised in toto with the submandibular gland. Other treatment approaches that may be attempted include laser surgery, cryosurgery, and injection of sclerosants, ligation of the feeding vessel, embolization, and corticosteroids. Due to the infrequency of its incidence, a high degree of suspicion is imperative for accurate diagnosis and prompt management. A differential diagnosis of vascular malformations should be considered for all soft, cystic lesions in the head and neck region and evaluated accordingly. 

Inaccurate and inadequate pre-operative evaluation of such lesions may prove catastrophic for the patient. The most commonly encountered cystic lesions in this region are usually lymphangiomas. The pre-operative groundwork and surgical approach to lymphangioma and haemangioendothelioma differ radically. Haemangioma excision requires meticulous planning for hemostasis and blood loss. Also, intra-operatively the dissection needs to be cautious and meticulous to avoid intractable hemorrhage, as it may obscure the field making dissection and recognition of anatomical landmarks very difficult. This is especially dangerous in the submandibular region due to the proximity of various vital vascular and neural elements. Cho et al observed that torrential hemorrhage occurred in patients in whom the surgeons were unable to ligate the feeding vessel (10). Aynali et al. (8) also made similar observations in their study. In very large and highly vascular lesions, pre-operative embolization may be attempted to reduce the vascularity of the lesion before the definitive surgical procedure. However, Kumar et al in their study observed that embolization did not confer any significant benefit in terms of intraoperative blood loss (11). 

## Conclusion

In conclusion, a differential diagnosis of haemangioma should always be considered by surgeons and radiologists alike for lesions with suspicious or indeterminate features, in this region. 
